# Mind the Gap: Inadequate Performance of Embolic Risk Scores in Infective Endocarditis

**DOI:** 10.1093/ofid/ofag280

**Published:** 2026-05-20

**Authors:** Nicoleta Ianculescu, Pierre Monney, Jana Epprecht, Georgios Tzimas, Michelle Frank, Lars Niclauss, Matthias Kirsch, Mathias Van Hemelrijck, Omer Dzemali, Benoit Guery, Barbara Hasse, Matthaios Papadimitriou-Olivgeris

**Affiliations:** Department of Cardiology, Lausanne University Hospital and University of Lausanne, Lausanne, Switzerland; Department of Cardiology, Lausanne University Hospital and University of Lausanne, Lausanne, Switzerland; Department of Infectious Diseases and Hospital Epidemiology, University Hospital Zurich and University of Zurich, Zurich, Switzerland; Department of Cardiology, Lausanne University Hospital and University of Lausanne, Lausanne, Switzerland; Department of Cardiology, University Hospital Zurich and University of Zurich, Zurich, Switzerland; Department of Cardiac Surgery, Lausanne University Hospital and University of Lausanne, Lausanne, Switzerland; Department of Cardiac Surgery, Lausanne University Hospital and University of Lausanne, Lausanne, Switzerland; Department of Cardiac Surgery, University Hospital Zurich and University of Zurich, Zurich, Switzerland; Department of Cardiac Surgery, University Hospital Zurich and University of Zurich, Zurich, Switzerland; Department of Cardiac Surgery, City Hospital of Zurich—Triemli, Zurich, Switzerland; Center for Experimental and Translational Cardiology, University of Zurich, Zurich, Switzerland; Infectious Diseases Service, Lausanne University Hospital and University of Lausanne, Lausanne, Switzerland; Department of Infectious Diseases and Hospital Epidemiology, University Hospital Zurich and University of Zurich, Zurich, Switzerland; Infectious Diseases Service, Lausanne University Hospital and University of Lausanne, Lausanne, Switzerland; Infectious Diseases Service, Hospital of Valais and Institut Central des Hôpitaux, Sion, Switzerland

**Keywords:** embolic events, infective endocarditis, prediction score, *Staphylococcus aureus*, vegetation

## Abstract

**Background:**

Embolic events (EEs) are frequent and clinically important complications of infective endocarditis (IE). Three scores have been developed to predict EEs: the *Embolic Risk French Calculator* (for EEs occurring after treatment initiation), and the *Italian Endocarditis Study* and *University of Campania “L. Vanvitelli” Napoli Score* (for EEs occurring before or after antimicrobial initiation). This study aimed to externally validate and compare the predictive performance of these scores.

**Methods:**

This is a multicenter retrospective study including adult patients diagnosed with IE between 2014 and 2024 at Lausanne University Hospital and University Hospital Zurich. Embolic events and IE were defined according to the 2023 International Society of Cardiovascular Infectious Diseases (ISCVID) Duke criteria. Scores were applied according to their respective populations, and performance was evaluated using sensitivity, specificity, and accuracy.

**Results:**

Among 1331 IE episodes, 674 (51%) experienced EEs, with 474 (36%) before and 348 (26%) after antimicrobial initiation. The Embolic Risk French Calculator, applied to 1201 valve-related IE episodes, identified 358 (30%) as high risk. Accuracy for predicting postantibiotic EEs was 66% (95% CI 64%–69%). The Italian Endocarditis Study score, applied to 1073 episodes with left-side IE, classified 563 (53%) as intermediate/high risk, with an overall accuracy of 60% (56%–62%). The University of Campania score, applied to 701 valve-related IE episodes, identified 341 (49%) as intermediate/high risk, with an accuracy of 63% (60%–67%).

**Conclusions:**

All 3 scores demonstrated limited predictive accuracy and frequently misclassified patients. More robust tools are needed to guide embolic risk stratification in clinical practice.

## Teaser

Among 1331 infective endocarditis episodes, the Embolic Risk French Calculator, Italian Endocarditis Study Score, and University of Campania “L. Vanvitelli” Napoli Score predicted embolic events with accuracies of 66% (64%–69%), 60% (56%–62%), and 63% (60%–67%), respectively.

## Graphical Abstract

**Graphical Abstract:**
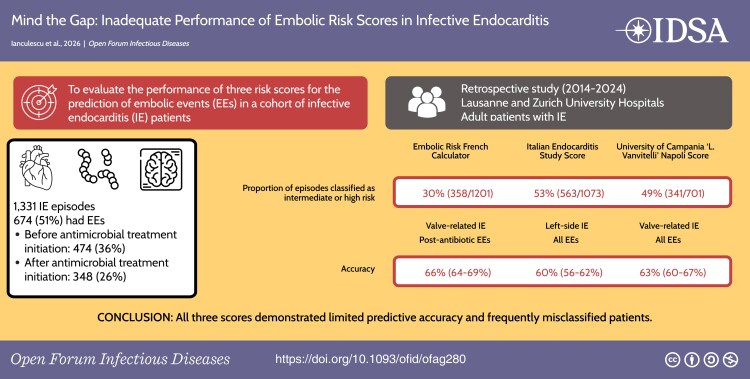
This graphical abstract is also available at Tidbit: https://tidbitapp.io/tidbits/mind-the-gap-inadequate-performance-of-embolic-risk-scores-in-infective-endocarditis-e66bd059-571b-4068-bb2d-c161fd582792.

Embolic events (EEs) are among the most frequent and clinically significant complications of infective endocarditis (IE), occurring in 20%–50% of cases and contributing substantially to morbidity and mortality [1–3]. While the initiation of antimicrobial therapy rapidly reduces the risk of new EEs embolization can still occur during treatment [3–6]. The presence of large vegetations, defined as ≥10 mm on the left side and ≥20 mm on the right side of the heart, is associated with a higher embolic risk and may indicate the need for early surgery, as recommended by the European Society of Cardiology (ESC) [7]. Identifying patients at high risk of EEs is critical for guiding surgical decisions. The most consistently reported predictors include large vegetations (≥10 mm), *Staphylococcus aureus* IE, and EEs occurring before antibiotic initiation [1–3, 8].

To facilitate risk stratification, 3 predictive scores have been developed. The *Embolic Risk French Calculator* [9], designed to predict posttreatment EEs, derived from a cohort of 565 patients with valve-related IE, includes 6 variables: age, diabetes mellitus, atrial fibrillation, *S aureus* IE, pretreatment EEs, and vegetation size. It was validated in 282 patients from the original cohort and in several external populations, including cohorts from France (n = 533), Japan (n = 166 and n = 52), Spain (n = 153), and the Philippines (n = 87) [9–14]. The *Italian Endocarditis Study Score* [15], designed to predict all EEs, developed from 1306 patients with left-side IE (LS-IE), uses 2 parameters: *S aureus* IE and vegetation size ≥13 mm. It stratifies patients into intermediate- and high-risk groups and has been externally validated in a French cohort (n = 533) [10, 15]. The *University of Campania “L. Vanvitelli” Napoli Score*, designed to predict all EEs, based on 715 patients, incorporates 5 predictors: *S aureus* IE, vegetation size ≥14 mm, D-dimer >747 ng/dL, C-reactive protein >67 mg/L, and splenomegaly (>12 cm) [16].

All 3 scores have consistently shown that patients classified as high or intermediate/high risk experience significantly more EEs than those at low risk. The aim of this study was to evaluate the performance of these 3 embolic risk scores in a multicenter cohort of IE patients.

## METHODS

### Study Design

This retrospective–prospective study was conducted at 2 Swiss tertiary care centers, Lausanne University Hospital (CHUV) and University Hospital Zurich (USZ), and included patients with IE from January 2014 to June 2024. Patients were drawn from existing institutional IE cohorts: CHUV's cohort of suspected IE cases and USZ's cohort of confirmed IE cases. Both centers included patients retrospectively from January 2014 to December 2017 and prospectively thereafter until June 2024. The study was approved by the relevant Swiss ethics committees (CER-VD 2017–02137, KEK-2014-0461, BASEC-2017-01140).

### Study Participants

Eligible participants were adults (≥18 years) with a diagnosis of IE and no documented objection to data use. Recurrent IE episodes occurring within 2 months of a prior episode were excluded.

From January 2018 onward, IE diagnoses were established by institutional Endocarditis Teams. For earlier cases, diagnosis was adjudicated by 2 experienced clinicians from each center (CHUV: M. P.- O., P. M.; USZ: B. H., M. F.), all of whom have served on their respective Endocarditis Teams since 2018. Classification into possible or definite IE was based on the 2023 International Society of Cardiovascular Infectious Diseases (ISCVID) Duke criteria [17]. At both institutions, infectious diseases consultation was mandatory for all IE cases. Clinical data were manually extracted from electronic health records, including demographics, comorbidities, imaging findings, microbiological results, EEs, surgical interventions, and pathology reports. All cases were reviewed by ID specialists.

### Definitions

Embolic events were defined according to ISCVID criteria and included peripheral arterial embolism, septic pulmonary emboli, visceral emboli (hepatic, renal, splenic), mycotic aneurysm, central nervous system complications (ischemic or hemorrhagic stroke, cerebral abscess), ocular (eg, conjunctival hemorrhages, retinal emboli, or endophthalmitis) and cutaneous manifestations (Janeway lesions or splinter hemorrhages) [17]. Embolic events were categorized as occurring either before or within 60 days after initiation of antimicrobial therapy. Events were considered symptomatic when associated with localizing symptoms (eg, focal neurological deficits, dyspnea, abdominal, or back pain). Cutaneous EEs were classified symptomatic, as they are detectable on clinical examination without the need of imaging. Imaging (thoracoabdominal or cerebral) was routinely performed in symptomatic patients, whereas in asymptomatic individuals, it was conducted at the discretion of the treating physicians and infectious diseases consultants. Cutaneous EEs were considered asymptomatic. The timing of symptomatic EEs was defined the onset of symptoms, while for asymptomatic events, the date of the radiologic diagnosis date was used. All EEs identified within 48 hours of presentation, defined as hospital admission for community-onset IE or first blood culture collection for nosocomial IE, were classified as occurring prior to antimicrobial treatment, irrespective of symptom status or timing of treatment initiation. Three embolic risk scores were subsequently applied to assess predictive performance.

1. The Embolic Risk French Calculator, designed to predict posttreatment EEs, was applied to valve-related IE cases; a predicted embolic risk of ≥7% at 14 days was considered high-risk [9].
2. The Italian Endocarditis Study Score, designed to predict all EEs, applied to LS-IE cases, classified patients with ≥1 point as intermediate or high-risk [15].
3. The University of Campania “L. Vanvitelli” Napoli Score, designed to predict all EEs, was applied to valve-related IE cases from the CHUV cohort only, as D-dimer levels and splenomegaly data were unavailable for USZ patients. A score ≥3 was considered intermediate or high-risk [16].

### Statistics

Statistical analyses were performed using SPSS version 26.0 (IBM Corp., Armonk, NY, USA). Categorical variables were expressed as frequencies and percentages and compared using the χ^2^ test or Fisher's exact test, as appropriate. Continuous variables were summarized using medians and interquartile ranges (IQRs) and compared using the Mann–Whitney *U* test due to nonnormal distribution of most variables. Missing values were assumed to reflect the absence of the respective clinical feature and were treated accordingly.

To evaluate the predictive performance of each embolic risk score, sensitivity, specificity, positive predictive value (PPV), negative predictive value (NPV), and overall accuracy were calculated, each with corresponding 95% confidence intervals (CIs). Receiver operating characteristic curves were generated to calculate the area under the curve (AUROC), assessing the discriminative ability of the 3 scores in predicting EEs. All statistical tests were 2-tailed, and a *P*-value of <.05 was considered statistically significant.

## RESULTS

**Figure 1.**
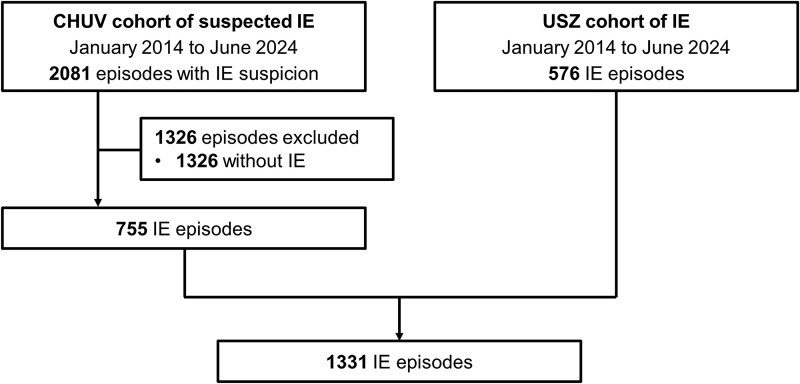
Flowchart of included episodes. CHUV, Lausanne University Hospital; IE, infective endocarditis; USZ, University Hospital Zurich.

Among 2657 episodes of suspected IE across both cohorts, 1331 episodes fulfilled inclusion criteria and were analyzed (Figure 1). Among the 1331 IE episodes, 1075 (81%) were classified as definite IE by the 2023 ISCVID Duke criteria. Of the 1331 IE episodes, 846 (64%) involved native valves, 366 (27%) prosthetic valves, and 197 (15%) cardiac implantable electronic device leads, with 92 (7%) episodes having multisite IE (Table 1). Based on each score's intended population, 1201 episodes of valve-related IE were evaluated using the Embolic Risk French Calculator, all 1073 LS-IE episodes were assessed using the Italian Endocarditis Study Score, and 701 valve-related IE episodes from the CHUV cohort were analyzed using the University of Campania “L. Vanvitelli” Napoli Score.

**Table 1.** Comparison of 1331 Infective Endocarditis Episodes With or Without Embolic Events Before or After Antimicrobial Treatment Initiation

	No Embolic Events (n = 657)	Embolic Events (n = 674)	*P*
Demographics and comorbidities			
Male sex, n (%)	485 (74)	502 (75)	.802
Age (years), median (IQR)	68 (52–77)	64 (50–73)	<.001
Age >60 y, n (%)	429 (65)	388 (58)	.004
Malignancy (solid organ or hematologic), n (%)	97 (14)	75 (11)	.050
Immunosuppression^a^, n (%)	47 (7)	50 (7)	.916
Diabetes mellitus, n (%)	120 (20)	148 (23)	.096
Chronic kidney disease (moderate or severe), n (%)	189 (29)	137 (20)	<.001
Obesity (body mass index ≥30 kg/m^2^), n (%)	129 (20)	137 (20)	.784
Chronic obstructive pulmonary disease, n (%)	75 (11)	60 (9)	.146
Congestive heart failure, n (%)	139 (21)	92 (14)	<.001
Atrial fibrillation, n (%)	203 (31)	163 (24)	.007
Cirrhosis, n (%)	79 (12)	85 (13)	.803
Charlson Comorbidity Index (points), median (IQR)	3 (1–6)	3 (1–5)	.001
Charlson Comorbidity Index >4, n (%)	253 (39)	199 (30)	.001
Setting of infection onset^a^	…	…	.002
Community-acquired, n (%)	487 (74)	547 (81)	…
Healthcare-associated, n (%)	74 (11)	65 (10)	…
Nosocomial, n (%)	97 (15)	62 (9)	…
Cardiac predisposing factors			
Intravenous drug use, n (%)	44 (7)	81 (12)	.001
Prosthetic valve, n (%)	225 (34)	181 (27)	.004
Prior endocarditis, n (%)	96 (15)	70 (10)	.020
CIED, n (%)	193 (29)	95 (14)	<.001
Microbiological data			
*S aureus*, n (%)	205 (31)	279 (41)	<.001
Coagulase-negative staphylococci, n (%)	51 (8)	51 (8)	.918
*Streptococcus* sp., n (%)	142 (22)	180 (27)	.035
*Enterococcus* sp., n (%)	110 (17)	66 (10)	<.001
Other Gram-positive, n (%)	28 (4)	21 (3)	.309
HACEK, n (%)	15 (2)	9 (1)	.220
Gram-negative other than HACEK, n (%)	38 (6)	28 (4)	.206
Fungi, n (%)	16 (2)	12 (2)	.449
Intracellular microorganisms, n (%)	5 (0.8)	11 (2)	.208
Culture-negative, n (%)	81 (12)	48 (7)	.002
Clinical manifestation			
Fever, n (%)	506 (77)	537 (80)	.258
Sepsis, n (%)	211 (32)	329 (49)	<.001
Immunological phenomena, n (%)	47 (7)	109 (16)	<.001
Native bone and joint infection, n (%)	77 (12)	87 (13)	.559
Septic arthritis, n (%)	45 (7)	51 (8)	.672
Spondylodiscitis, n (%)	37 (6)	44 (7)	.567
Laboratory			
C-reactive protein (mg/L), median (IQR)^b^	97 (46–180)	149 (70–253)	<.001
D-dimers (ng/mL), median (IQR)^c^	3583 (1956–7534)	5538 (2991–13740)	.976
Imaging data			
Vegetation, n (%)	399 (61)	543 (81)	<.001
Vegetation ≥10mm, n (%)	182 (28)	356 (53)	<.001
Abscess, n (%)	77 (12)	159 (24)	<.001
Perforation, dehiscence of prosthesis, n (%)	72 (11)	93 (14)	.134
Aneurysm, pseudoaneurysm, fistula, n (%)	23 (4)	24 (4)	1.000
Leaflet thickening, n (%)	74 (11)	83 (12)	.610
Significant new valvular regurgitation, n (%)	169 (26)	214 (32)	.016
Type of infective endocarditis			
Valve related	555 (85)	646 (96)	<.001
Aortic, n (%)	317 (48)	332 (49)	.742
Mitral, n (%)	223 (34)	321 (48)	<.001
Tricuspid, n (%)	42 (6)	70 (10)	.010
Pulmonary, n (%)	25 (4)	15 (2)	.108
Native valve, n (%)	361 (55)	485 (72)	<.001
Prosthetic valve, n (%)	195 (30)	171 (25)	.086
CIED-lead related, n (%)	136 (21)	61 (9)	<.001

HACEK includes *Haemophilus* species (eg, *Haemophilus parainfluenzae*, *H aphrophilus*), *Aggregatibacter* species (eg, *Aggregatibacter actinomycetemcomitans*), *Cardiobacterium hominis*, *Eikenella corrodens*, *Kingella* species (eg, *Kingella kingae*).

CIED, cardiac implantable electronic device; IQR, interquartile range.

^a^Comparison between community bacteremia and both healthcare-associated and nosocomial.

^b^Available in 1215 of 1331 (91%) episodes.

^c^Available in 115 of 701 episodes (16%) of the CHUV patients.

Transthoracic echocardiography was performed in 1206 episodes (91%), transesophageal echocardiography in 926 (70%), ^18^F-Fluorodeoxyglucose Positron Emission Tomography/Computed Tomography (^18^F-FDG PET/CT) in 259 (19%), and cardiac CT in 65 (5%). In the CHUV cohort (n = 755), thoracoabdominal imaging (CT, MRI, or ^18^F-FDG PET/CT was performed in 618 (82%) episodes and cerebral imaging (CT or MRI) in 480 (64%).

Overall, 674 (51%) of 1331 episodes were associated with EEs. Embolic events occurred before antimicrobial treatment initiation in 474 (36%) episodes, including 263 cerebral and 345 noncerebral events. Embolic events occurred after antimicrobial treatment initiation in 348 (26%) episodes, with 253 cerebral and 154 noncerebral events (Table 2). Supplementary Figure 1 presents the Kaplan–Meier analysis comparing EE rates after antimicrobial treatment initiation between episodes with and without prior EEs (log-rank test *P* < .001). Symptomatic EEs were identified in 575 (43%) episodes, occurring before antimicrobial initiation in 423 (32%) and after in 275 (21%) episodes.

**Table 2.** Comparison of 1331 Infective Endocarditis Episodes With or Without Embolic Events After Antimicrobial Treatment Initiation

	No Embolic Events (n = 983)	Embolic Events (n = 348)	*P Values*
Demographics and comorbidities			
Male sex, n (%)	719 (73)	268 (77)	.176
Age (years), median (IQR)	66 (51–76)	65 (52–73)	.207
Age >60 y, n (%)	607 (62)	210 (60)	.004
Malignancy (solid organ or hematologic), n (%)	131 (13)	41 (12)	.515
Immunosuppression^a^, n (%)	70 (7)	27 (8)	.719
Diabetes mellitus, n (%)	192 (20)	95 (27)	.003
Chronic kidney disease (moderate or severe), n (%)	250 (25)	76 (22)	.192
Obesity (body mass index ≥30 kg/m^2^), n (%)	185 (19)	81 (23)	.086
Chronic obstructive pulmonary disease, n (%)	104 (11)	31 (9)	.410
Congestive heart failure, n (%)	181 (18)	50 (14)	.099
Atrial fibrillation, n (%)	271 (28)	95 (27)	.944
Cirrhosis, n (%)	105 (11)	59 (17)	.003
Charlson Comorbidity Index (points), median (IQR)	3 (1–6)	3 (1–5)	.264
Charlson Comorbidity Index >4, n (%)	350 (26)	102 (29)	.035
Setting of infection onset^a^			<.001
Community-acquired, n (%)	740 (75)	294 (85)	…
Healthcare-associated, n (%)	108 (11)	31 (9)	…
Nosocomial, n (%)	136 (14)	23 (7)	…
Cardiac predisposing factors			
Intravenous drug use, n (%)	89 (9)	36 (10)	.521
Prosthetic valve, n (%)	303 (31)	103 (30)	.685
Prior endocarditis, n (%)	127 (13)	39 (11)	.451
CIED, n (%)	238 (24)	50 (14)	<.001
Microbiological data			
*S aureus*, n (%)	334 (34)	150 (43)	.003
Coagulase-negative staphylococci, n (%)	78 (8)	24 (87)	.639
*Streptococcus* sp., n (%)	224 (23)	98 (28)	.049
*Enterococcus* sp., n (%)	148 (15)	28 (8)	.001
Other Gram-positive, n (%)	40 (4)	9 (3)	.248
HACEK, n (%)	20 (2)	4 (1)	.355
Gram-negative other than HACEK, n (%)	56 (6)	10 (3)	.043
Fungi, n (%)	23 (2)	5 (1)	.389
Intracellular microorganisms, n (%)	9 (0.9)	7 (2)	.147
Culture-negative, n (%)	106 (11)	23 (7)	.026
Clinical manifestation			
Fever, n (%)	758 (77)	285 (82)	.069
Sepsis, n (%)	365 (37)	175 (50)	<.001
Immunological phenomena, n (%)	101 (10)	55 (16)	.009
Native bone and joint infection, n (%)	109 (11)	55 (16)	.029
Native bone and joint infection after antimicrobial treatment initiation, n (%)	20 (2)	21 (6)	<.001
Septic arthritis, n (%)	64 (7)	32 (9)	.116
Spondylodiscitis, n (%)	55 (6)	27 (8)	.155
Embolic event before antimicrobial treatment, n (%)	317 (32)	157 (45)	<.001
Cerebral embolic events, n (%)	184 (19)	79 (23)	.117
Noncerebral embolic events, n (%)	161 (16)	85 (24)	.001
Laboratory			
C-reactive protein (mg/L), median (IQR)^b^	222 (52–204)	166 (74–268)	<.001
D-dimers (ng/mL), median (IQR)^c^	4930 (2750–11956)	5539 (2476–10192)	<.001
Imaging data			
Vegetation, n (%)	659 (67)	283 (81)	<.001
Vegetation ≥10mm, n (%)	356 (36)	182 (52)	<.001
Abscess, n (%)	144 (15)	92 (26)	<.001
Perforation, dehiscence of prosthesis, n (%)	121 (12)	44 (13)	.850
Aneurysm, pseudoaneurysm, fistula, n (%)	34 (4)	13 (4)	.866
Leaflet thickening, n (%)	110 (11)	47 (14)	.247
Significant new valvular regurgitation, n (%)	277 (28)	105 (31)	.449
Type of infective endocarditis			
Valve related	864 (88)	337 (97)	<.001
Aortic, n (%)	464 (47)	185 (53)	.061
Mitral, n (%)	376 (38)	168 (48)	.001
Tricuspid, n (%)	84 (9)	28 (8)	.823
Pulmonary, n (%)	36 (4)	4 (1)	.017
Native valve, n (%)	599 (61)	247 (71)	.001
Prosthetic valve, n (%)	270 (28)	96 (28)	1.000
CIED-lead related, n (%)	169 (17)	27 (8)	<.001

HACEK includes *Haemophilus* species (eg, *H parainfluenzae, H aphrophilus*), *Aggregatibacter* species (eg, *A actinomycetemcomitans*), *C hominis, E corrodens, Kingella* species (eg, *K kingae*).

CIED, cardiac implantable electronic device, IQR, interquartile range.

^a^Comparison between community bacteremia and both healthcare-associated and nosocomial.

^b^Available in 1215 of 1331 (91%) episodes.

^c^Available in 115 of 701 episodes (16%) of the CHUV patients.

### Embolic Risk French Calculator

Among the 1201 valve-related IE episodes, 337 (28%) experienced EEs after the start of antimicrobial therapy, of which 267 (79%) were symptomatic. Applying the Embolic Risk French Calculator identified 358 (30%) as high-risk (predicted embolic risk ≥7% at day 14) (Table 3). Among these high-risk episodes, 145 (41%) had posttreatment EEs (symptomatic or asymptomatic), compared to 192 (23%) among the 843 low-risk episodes. The score's sensitivity was 43% (95% CI 38%–49%), specificity 75% (72%–78%), and overall accuracy 66% (64%–69%) (Table 4). The AUROC for the score using the cutoff of ≥7% predicted embolic risk at day 14 was 0.58 (0.54–0.62) **(**Supplementary Figure 2*A*). Among the 358 high-risk episodes, 116 (32%) developed posttreatment symptomatic EEs, compared to 151 (18%) among the 843 low-risk episodes. The performance of the Embolic Risk French Calculator was similar when restricted to the prediction of posttreatment symptomatic EEs (Supplementary Table 1).

**Table 3.** Classifications based on the Different Scores

	No Embolic Events	Embolic Events	*P*
Embolic Risk French Calculator	After antimicrobial treatment initiation		
Valve-related infective endocarditis	n = 864	n = 337	
Age (years), median (IQR)	65 (49–76)	65 (52–73)	.688
Diabetes mellitus, n (%)	156 (18)	91 (27)	.001
Embolic events before antimicrobial treatment initiation, n (%)	302 (35)	156 (46)	<.001
Atrial fibrillation, n (%)	207 (24)	89 (26)	.372
Vegetation length			<.001
No vegetation, n (%)	339 (39)	92 (27)	…
Vegetation ≤10mm, n (%)	242 (28)	83 (25)	…
Vegetation >10mm, n (%)	283 (33)	162 (48)	…
*S aureus*, n (%)	283 (33)	144 (42)	.002
Cumulative incidence of embolic events at day 14 (%), median (IQR)	4 (2–7)	6 (3–11)	<.001
High risk of embolic event at day 14 (>7%), n (%)	213 (25)	145 (43)	<.001
Italian study on Endocarditis score	Before or after antimicrobial treatment initiation		
Left-side infective endocarditis	n = 493	n = 580	
Vegetation ≥13mm, n (%)	76 (15)	220 (38)	<.001
*S aureus*, n (%)	144 (29)	209 (36)	<.001
Points, median (IQR)	0 (0–1)	1 (0–1)	<.001
Risk classification	…	…	<.001
Low (0 points), n (%)	284 (58)	226 (39)	…
Intermediate (1 point), n (%)	198 (40)	279 (48)	…
High (2 points), n (%)	11 (2)	75 (13)	…
University of Campania “L. Vanvitelli” Napoli score	Before or after antimicrobial treatment initiation		
Valve-related infective endocarditis (CHUV cohort)	n = 314	n = 387	
*S aureus*, n (%)	98 (31)	165 (43)	.002
D-dimer >747 ng/dL, n (%)	26 (8)	75 (19)	<.001
C-reactive protein >67 mg/l, n (%)	189 (60)	274 (71)	.004
Vegetation ≥14 mm, n (%)	44 (14)	147 (38)	<.001
Splenomegaly (longitudinal diameter >12 cm), n (%)	14 (5)	56 (15)	<.001
Points, median (IQR)	2 (1–3)	3 (2–4)	<.001
Risk classification			<.001
Low (0–2 points), n (%)	209 (67)	151 (39)	…
Intermediate (3–5 points), n (%)	104 (33)	201 (52)	…
High (6–8 points), n (%)	1 (0.3)	35 (9)	…

IQR, interquartile range.

**Table 4.** Performance of the Three Scores in Predicting Embolic Events in Patients With IE

	Sensitivity % (95% CI)	Specificity % (95% CI)	PPV % (95% CI)	NPV % (95% CI)	Accuracy % (95% CI)
Embolic Risk French Calculator^a^ (1201 episodes of valve-IE)	43 (38–49)	75 (72–78)	41 (37–45)	77 (75–79)	66 (64–69)
Italian study on Endocarditis score^b^ (1073 episodes left-side IE)	61 (57–65)	58 (53–62)	63 (60–66)	56 (53–59)	60 (56–62)
University of Campania “L. Vanvitelli” Napoli score^c^ (701 episodes of valve-IE)	61 (56–66)	67 (61–72)	69 (65–73)	58 (54–62)	63 (60–67)

IE, infective endocarditis; NPV, negative predictive value; PPV, positive predictive value.

^a^After antimicrobial treatment initiation; high risk of embolic event at day 14 (>7%).

^b^Before or after antimicrobial treatment initiation; intermediate or high risk (≥1 point).

^c^Before or after antimicrobial treatment initiation; intermediate or high risk (≥3 points).

### Italian Study on Endocarditis Score

Applying the Italian Endocarditis Study Score to 1073 episodes of LS-IE, 563 (53%) were categorized as intermediate or high risk (≥1 point) (Table 3). Embolic events occurred in 354 (63%) of these episodes, compared to 226 (44%) among the 510 low-risk cases. The score demonstrated a sensitivity of 61% (95% CI, 57%–65%), specificity 58% (53%–62%), and accuracy 60% (56%–62%) (Table 4). The AUROC for the score using the cutoff of ≥1 point was 0.59 (0.56–0.63) (Supplementary Figure 2*B*). Symptomatic EEs occurred in 304 (54%) of the 563 intermediate- or high-risk episodes, compared to 196 (38%) among the 510 low-risk cases. The performance of the Italian study on Endocarditis score was similar when restricted to the prediction of symptomatic EEs (Supplementary Table 1).

### University of Campania “L. Vanvitelli” Napoli Score

Among 701 valve-related IE episodes in the CHUV cohort, 387 (55%) were complicated by EEs, of which 300 (43%) were symptomatic. Applying the Napoli score identified 341 (49%) as intermediate or high risk (≥3 points) (Table 3). In this group, 236 patients (69%) developed EEs (symptomatic or asymptomatic), compared to 151 (42%) among the 360 low-risk patients. The score achieved a sensitivity of 61% (95% CI, 56–66), specificity 67% (61%–72%), and accuracy 63% (60%–67%) (Table 4). The AUROC for the score using the cutoff of ≥3 points was 0.64 (0.60–0.68) (Supplementary Figure 2*C*). Symptomatic EEs occurred in 195 (57%) of the 341 intermediate- or high-risk episodes, compared to 105 (29%) among the 360 low-risk cases. The performance of the University of Campania “L. Vanvitelli” Napoli score was similar when restricted to the prediction of symptomatic EEs (Supplementary Table 1).

Supplementary Table 2 presents the performance of the 3 scores for predicting EEs in episodes with LS-IE.

## DISCUSSION

In this large multicenter study, we found that the 3 currently available embolic risk scores for IE demonstrated limited accuracy in predicting EEs, particularly those occurring after the initiation of antimicrobial therapy. While each score was able to identify patients with a higher likelihood of EEs, a substantial proportion of embolic complications still occurred in patients classified as low risk.

EEs were remarkably frequent in our cohort; over half of the IE episodes experienced at least one EE, and 26% developed an EE after starting antibiotics, higher than previously reported [1]. This elevated rate likely reflects our cohort's extensive use of thoracoabdominal and cerebral imaging, which included asymptomatic patients and likely increased detection of silent EEs [18–20]. Consistent with previous studies, EEs were more frequent in IE caused by *S aureus* and in patients with large vegetations [1–3, 8].

The Embolic Risk French Calculator identified a higher proportion of postantimicrobial therapy EEs in high-risk patients (41%) compared to low-risk patients (23%), reproducing findings from earlier validations [9–14]. However, 57% of EEs still occurred in low-risk cases, indicating substantial misclassification. While the score includes 3 well-established predictors, such as large vegetations, *S aureus* IE, and pretreatment EEs, it also incorporates variables such as age, diabetes, and atrial fibrillation, which have shown inconsistent associations with EEs across studies [11–14]. In our cohort, as in others, age and atrial fibrillation were not significantly associated with EEs, and although diabetes mellitus was more common among episodes with EEs, this finding has not been replicated consistently [11–14]. These observations suggest that inclusion of weak or nonindependent predictors may reduce the score's discriminative performance.

The Italian Endocarditis Study Score, despite its simplicity and reliance on 2 of the most robust predictors (*S aureus* and large vegetations ≥13 mm), classified one-third of patients developed EEs as low-risk [15]. The widespread presence of these risk factors among IE cases reduces the score's ability to differentiate between patients truly at high versus low risk [21, 22].

The University of Campania “L. Vanvitelli” Score showed reduced performance compared to its original derivation study, with lower sensitivity (61% vs 83%) and NPV (58% vs 82%) [16]. This score includes C-reactive protein >67 mg/L, splenomegaly, and elevated D-dimer levels, variables that are subject to detection bias. For instance, splenomegaly was typically diagnosed only when abdominal imaging was performed, and elevated D-dimers often prompted additional imaging for pulmonary embolism, increasing the likelihood of identifying otherwise asymptomatic EEs. Although CRP >67 mg/L was more frequent in episodes with EEs (71% vs 60%), this difference was less pronounced than in the original study (61% vs 43%) [16].

Beyond the intrinsic limitations of these scores, several broader issues should be considered. First, while identifying episodes at high risk of postantibiotic EEs is clinically valuable, since it may influence surgical decisions, predicting EEs that occur before initiation of antimicrobial therapy is less impactful [7]. Symptomatic EEs before treatment are usually detected since the presence of symptoms leads to performance of imaging, and identifying asymptomatic events prior to treatment rarely alters management [18, 19, 23, 24]. As shown in prior studies, while symptomatic EEs are associated with mortality [23], asymptomatic EEs do not affect clinical decision-making and do not impact outcome [18, 19, 23, 24]. Second, the lack of consistency in vegetation size thresholds across the scores (ranging from ≥10 to ≥14 mm) complicates clinical application [9, 15, 16]. A uniform cutoff of ≥10 mm, as recommended by ESC guidelines for considering surgery to prevent embolism, would enhance consistency [7]. Third, the Embolic Risk French Calculator and the University of Campania “L. Vanvitelli” score involve complex calculations [9, 16], limiting their use in day-to-day clinical practice. Finally, none of the evaluated scores accounts for valve surgery, a key intervention shown to reduce embolic risk when used alongside antibiotic therapy [3, 25].

This study has several strengths, including a large cohort drawn from 2 expert centers with consistent diagnostic and management practices, including systematic infectious diseases consultation and multidisciplinary IE management. Importantly, the proportion of patients undergoing thoracoabdominal and cerebral imaging was high, even in the absence of symptoms, enhancing the detection of asymptomatic events. Unlike the studies used to establish risk scores [9, 15, 16], we provide a detailed account of the proportion of patients who underwent imaging for EE detection. Additionally, our analysis was based on the 2023 ISCVID Duke criteria [17], which offer a clear and clinically relevant definition of EEs, as opposed to broader classifications like those in the ESC guidelines [7], which include hematogenous complications such as spondylodiscitis and septic arthritis. We excluded these conditions due to their likely pathophysiology of bacteremic seeding rather than embolism from valvular vegetations [26–29]. Furthermore, we performed a subgroup analysis restricted to episodes with LS-IE, as these patients more frequently undergo surgery for the prevention of EEs, whereas right-sided IE is predominantly managed medically [7, 30].

Limitations include the study's observational design and restriction to a single healthcare system, which may affect generalizability to settings with less access to specialist care [7, 17]. Moreover, although imaging was widely performed, not all patients underwent complete thoracoabdominal or cerebral imaging, potentially leading to underestimation of EEs in some cases [26–29]. Furthermore, we evaluated both symptomatic and asymptomatic EEs. Although these entities differ in their clinical impact, both are relevant for evaluating the predictive performance of embolic risk scores. Restricting the analysis to episodes with symptomatic EEs yielded comparable results across all scores, consistent with those observed for overall EE prediction and underscoring their limited discriminative capacity. Moreover, the Embolic Risk French Calculator was originally derived from episodes classified as definite IE according to the 2000 Duke criteria, whereas the University of Campania “L. Vanvitelli” Napoli Score included cases defined by the 2000 or 2015 Duke criteria. Both versions have since been superseded by updated criteria with improved diagnostic performance [7, 17, 27, 30–33]. This heterogeneity in case definitions across derivation and validation cohorts may have influenced the observed predictive performance of this study. In the absence of a universally accepted reference standard for IE, case ascertainment in the present study relied on adjudication by multidisciplinary Endocarditis Teams or, prior to its implementation, by experienced clinicians with full access to clinical, microbiological, imaging, and surgical data. While 81% of cases fulfilled criteria for definite IE according to the 2023 ISCVID Duke criteria, we intentionally retained episodes classified as possible IE when they were clinically managed as IE. Although this approach introduces a degree of subjectivity and may have contributed to the modest discriminative performance of the evaluated scores, it reflects real-world clinical decision-making and aligns with current recommendations supporting multidisciplinary assessment [7]. Lastly, D-dimers were measured in only 15% of IE patients, illustrating the limited generalizability of the score in settings where D-dimer testing is not routinely performed [16].

In conclusion, we observed a high rate of EEs in patients with IE, particularly following the initiation of antimicrobial therapy. Although all 3 scores identified groups with elevated embolic risk, their overall predictive performance was limited. A substantial proportion of EEs occurred in patients classified as low risk. These findings underscore the need for a new, more accurate and clinically applicable risk model that focuses on predicting postantibiotic embolic complications. Future research should also assess whether early surgical intervention in high-risk patients, identified using such a model, can improve clinical outcomes.

## Supplementary Material

ofag280_Supplementary_Data

## Supplementary Data

Supplementary materials are available at *Open Forum Infectious Diseases* online. Consisting of data provided by the authors to benefit the reader, the posted materials are not copyedited and are the sole responsibility of the authors, so questions or comments should be addressed to the corresponding author.
