# Supplementary material for: Mind the Gap: Inadequate Performance of Embolic Risk Scores in Infective Endocarditis
Source: Open Forum Infect Dis. 2026 May 20;13(5):ofag280. doi: 10.1093/ofid/ofag280 (PMC13214560; doi:10.1093/ofid/ofag280)
Supplement: ofag280_Supplementary_Data [file ofag280_supplementary_data.pdf]

Supplementary Figure 1. Kaplan-Meier analysis comparing embolic event rates after antimicrobial treatment initiation between episodes with and without prior embolic events (log-rank test *P*<0.001).

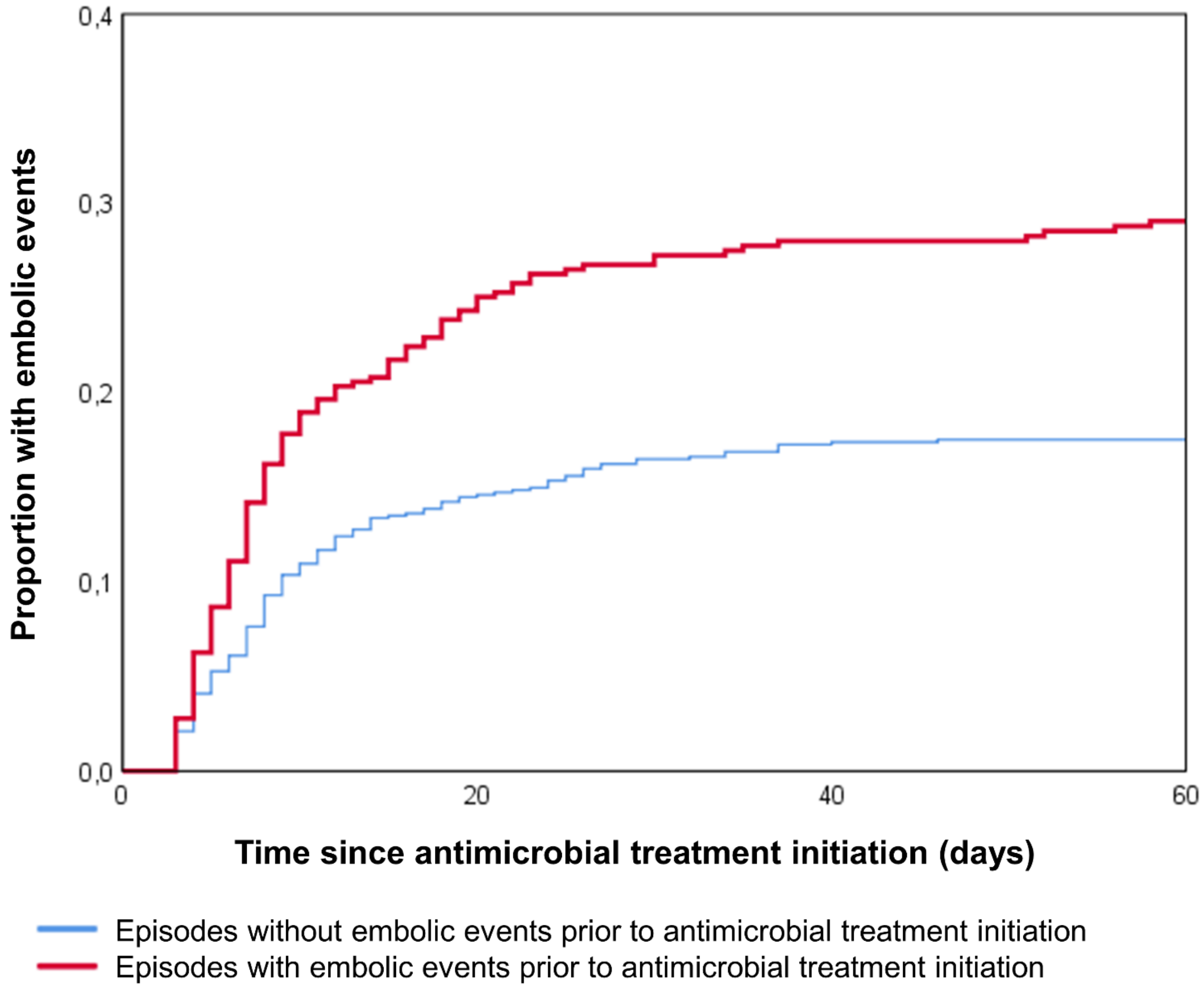

Supplementary Figure 2. Area under the receiver operating characteristic (AUROC) curves of **A)** the Embolic Risk French Calculator among 1,201 episodes of valve-related infective endocarditis (IE) for detecting embolic events after the start of antimicrobial therapy; **B)** the Italian Study on Endocarditis score among 1,072 episodes of left-side IE for detecting embolic events before and after the start of antimicrobial therapy; and **C)** the University of Campania "L. Vanvitelli" (Napoli) score among 701 episodes of valve-related IE for detecting embolic events before and after the start of antimicrobial therapy.

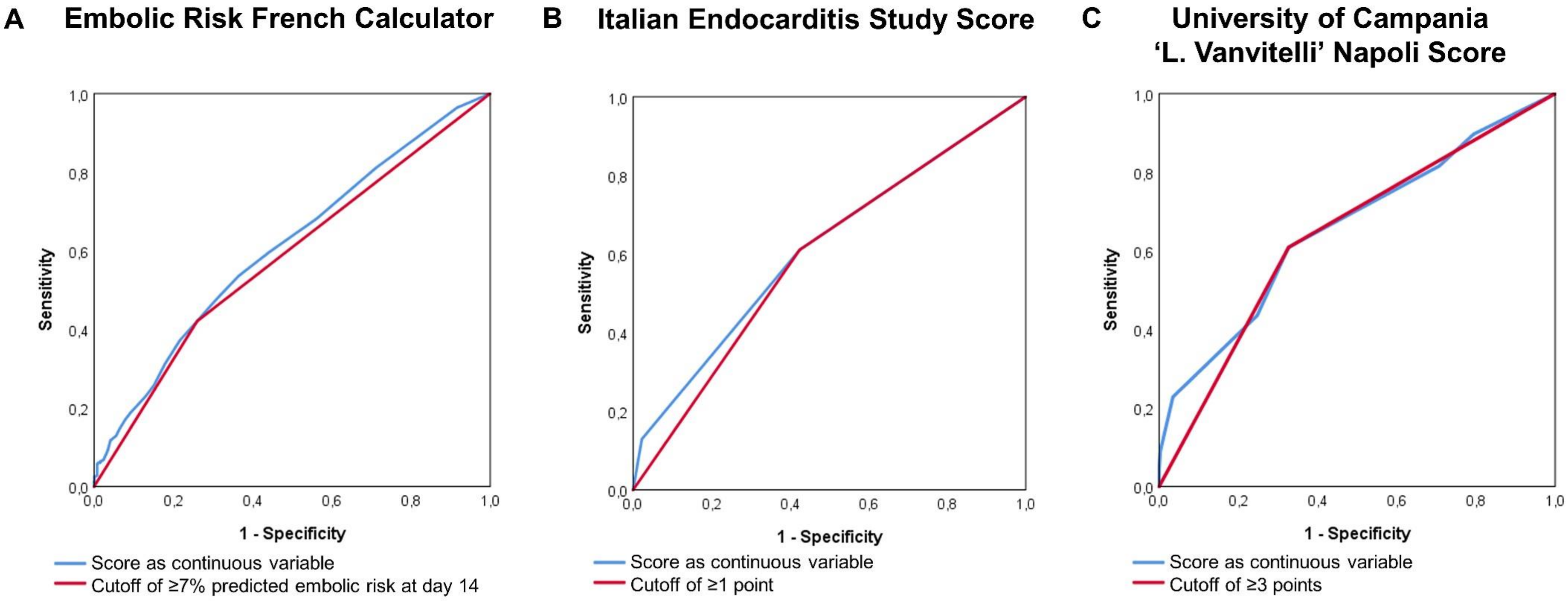

Supplementary Table 1. Performance of the three scores in predicting symptomatic embolic events in patients with IE

	**Sensitivity % (95% CI)**	**Specificity % (95% CI)**	**PPV % (95% CI)**	**NPV % (95% CI)**	**Accuracy % (95% CI)**
Embolic Risk French Calculator[a] (1201 episodes of valve-IE)	43 (37-50)	74 (71-77)	32 (29-36)	82 (80-84)	67 (65-70)
Italian study on Endocarditis score[b] (1073 episodes of left-side IE)	61 (56-65)	55 (51-58)	54 (51-57)	62 (58-65)	58 (55-61)
University of Campania ‘L. Vanvitelli’ Napoli score[c] (701 episodes of valve-IE)	65 (59-70)	64 (59-68)	57 (53-61)	71 (67-74)	64 (61-68)

IE: infective endocarditis; NPV: negative predictive value; PPV: positive predictive value

[a]after antimicrobial treatment initiation; high risk of embolic event at day 14 (>7%)

[b]before or after antimicrobial treatment initiation; intermediate or high risk (≥1 point)

[c]before or after antimicrobial treatment initiation; intermediate or high risk (≥3 points)

Supplementary Table 2. Performance of the three scores in predicting symptomatic embolic events in patients with left-side IE

	Sensitivity % (95% CI)	Specificity % (95% CI)	PPV % (95% CI)	NPV % (95% CI)	Accuracy % (95% CI)
Embolic Risk French Calculator[a] (1073 episodes of left-side IE)	43 (37-49)	75 (72-78)	41 (37-46)	76 (74-78)	66 (63-69)
Italian study on Endocarditis score[b] (1073 episodes of left-side IE)	61 (57-65)	58 (53-62)	63 (60-66)	56 (53-59)	60 (56-62)
University of Campania 'L. Vanvitelli' Napoli score[c] (633 episodes of left-side IE)	58 (53-63)	68 (62-73)	69 (65-73)	57 (53-60)	62 (59-66)

IE: infective endocarditis; NPV: negative predictive value; PPV: positive predictive value

[a]after antimicrobial treatment initiation; high risk of embolic event at day 14 (>7%)

[b]before or after antimicrobial treatment initiation; intermediate or high risk (≥1 point)

[c]before or after antimicrobial treatment initiation; intermediate or high risk (≥3 points)
